# Gauging support for autonomous delivery drones powered by artificial intelligence: A public survey in Singapore

**DOI:** 10.1371/journal.pone.0350394

**Published:** 2026-06-10

**Authors:** Justin C. Cheung, Shirley S. Ho

**Affiliations:** 1 Wee Kim Wee School of Communication and Information, Nanyang Technological University, Singapore, Singapore; 2 Campus for Research Excellence and Technological Enterprise, Singapore, Singapore; Providence University, TAIWAN

## Abstract

There has been emerging empirical evidence supporting the role of artificial intelligence (AI) knowledge in public support for AI. These findings stand in sharp contrast to the long-standing cognitive miser view in determining technological acceptance. In this study, by contrasting models of knowledge deficit against cognitive miser using data from a large-scale public opinion survey about autonomous delivery drones in Singapore (*N* = 1,002), we investigated whether knowledge and various heuristic cues were associated with AI support. Our results revealed that objective knowledge was not a direct determinant of support, while various heuristic cues such as propensity to trust in AI, news media attention, perceived source credibility in scientists and authorities, as well as risk and benefit perceptions presented significant associations. Mediation analyses further revealed knowledge was indirectly associated with support through heuristic cues such as source credibility perceptions as well as risk and benefit perceptions. Our findings were largely consistent with the cognitive miser model in that AI acceptance is immediately associated with cognitive heuristics rather than knowledge. Theoretical and practical implications are discussed.

## Introduction

In 2017, 65% of the American public believed that most deliveries in major cities would likely be carried out by drones within twenty years [1]. Nine years on, drone research is bringing that vision much closer to reality. Drones are at present certified to conduct postal delivery services in some densely populated urban areas [2]. There has also been significant development in testing autonomous delivery drone systems around the world [3,4]. These drones, powered by artificial intelligence (AI), are capable of enacting autonomous decision-making through real-time data collection and flight trajectory planning [5]. Recently, researchers have pointed to the promises of a “drone-to-everything” concept, which sets out to leverage on vigorous and seamless connectivity and coordination between a fleet of drones, ground devices, cloud servers, and human operators [6], thereby yielding significant benefits in an inter-connected technologically advanced urban environment [7]. Against this backdrop, we are motivated to investigate key determinants of public acceptance of AI-powered autonomous delivery drones, defined as “vehicles in the air which can operate fully autonomously with AI technology and without human intervention in executing the flight mission for the purpose of goods delivery from location to location.” This is a lay definition conceptualized with engineering scientists working on AI and drones which has received some empirical validation in similar technological contexts [8].

In gauging public opinion of emerging AI technologies such as drones, some empirical studies have adopted theoretical frameworks such as the extended unified theory of acceptance and use of technology (UTAUT2) [9] in understanding use and support for autonomous “taxi” drones [10], online AI assistants [11], and generative AI [12]. The UTAUT2, and similar frameworks it incorporates, captures public perceptions of various cognitive and affective responses to technological applications. Lately, however, much research has reported the promising value of AI knowledge [13], AI literacy [14], perceived AI knowledge [15], and domain-specific objective knowledge of autonomous vehicles [16] on AI acceptance. In this vein, much empirical research has also reported public knowledge levels on AI [17] as well as specific applications such as drones [18]. If there was substantial value in the knowledge variable, then, the primary objective of communication efforts would be to supply the public with appropriate scientific information.

What, then, determines acceptance in AI-powered delivery drones? Emerging empirical observations on AI knowledge are aligned with the long-standing knowledge deficit model which suggests that scientific knowledge is a prominent factor in predicting technological acceptance [19]. Yet, tension arises when the cognitive miser model is considered. The cognitive miser perspective suggests that people are inherently motivated to conserve mental efforts and, for this reason, technological acceptance hinges on cognitive heuristics rather than the more cognitively-demanding scientific knowledge [20]. This view has, likewise, received robust support as seen, for example, in empirical research on nanotechnology [21,22], as well as meta-analytic reports on nuclear energy [23]. In this study, we set out to replicate prior studies contrasting these theoretical perspectives in understanding public opinion of emerging technologies [24,25] and, more concretely, investigate how knowledge and various heuristic cues regress onto the outcome variable of AI support. By examining this issue in a novel and pressing AI context of autonomous delivery drones, we aim to add to the literature by further understanding how, in theory, knowledge and heuristic cues are associated with technological acceptance. These observations will not only provide new data on public opinion toward delivery drones, but also contribute to offering an answer to the aforementioned theoretical conflicts. In the face of an impending wave of AI-powered autonomous delivery drone operations, our findings will likewise practically inform policymakers and science communication practitioners in designing appropriate AI governance and communication strategies.

## Literature review

### AI, drones, and Singapore

Singapore has launched a national strategy outlining how AI is applied in different fields [26]. Envisioned as early as 2019, that strategy ensures that the country remains at the forefront of AI research and development, and was recently expanded to nurture engineering talents, invest in AI education, as well as preparing the future economy through utilizing the potentials of AI [27]. Singapore is also actively developing drone technologies. For example, it has tested food delivery drones [28] and developed drones capable of inspecting building facades with algorithms [29]. Against this backdrop, large-scale public surveys have found Singaporeans to be rather favorable of AI [30] and delivery drone technology [18]. Further, the Singaporean public appeared to demonstrate a reasonable level of familiarity with drone technologies, despite their knowledge levels not necessarily being in-depth [18].

### Knowledge deficit vis-à-vis cognitive miser models

We draw on two prominent theoretical frameworks in the science and technology studies field to develop our study. These two frameworks delineate divergent perspectives concerning public opinion in science and technology, namely the *knowledge deficit* model [19], and the *cognitive miser* model [20]. The underlying assumption of the knowledge deficit view is that public opposition to science and technology is attributed to a deficit of relevant technoscientific knowledge, and accordingly, increased knowledge is expected to promote support. The cognitive miser model, in contrast, assumes that individuals are intrinsically predisposed to conserve mental resources, thereby basing their judgements of technologies on easily-accessible heuristic cues rather than cognitively-demanding knowledge. Conceptually, knowledge “is represented in memory in terms of cross-links between cognitive units, complex hierarchical structures, and tangled multilevel interconnections” (p. 362) [31]. It is characterized by “a highly valued state in which a person is in cognitive contact with reality” (p. 92) [32]. In the science domain, Ho and Chuah have delineated knowledge in terms of understanding of the *general* science, and *domain-specific* (i.e., application-specific) knowledge, which contains *content* (i.e., scientific principles) and *contextual* (i.e., situational application) knowledge [33]. Heuristics, on the other hand, are “simple decision procedures that require little information processing” (p. 1477) [34]. Simply put, they are mental shortcuts that offer quick and low-effort decision-making.

The two models have long been contrasted against each other in public opinion studies of different technological applications, including embryonic stem cell research [35], nanotechnology [36], nuclear energy [23], autonomous vehicles [24], and alternative aquafeed [25]. This line of research has suggested that heuristics such as value predispositions tended to be relatively more robust predictors of acceptance as compared to knowledge, that is, even when knowledge presented significant associations with desirable outcomes, the effect was typically rather small [37]. Despite the substantial empirical evidence that supports the cognitive miser perspective, it is, in our view, imperative to contrast these theoretical accounts in the context of AI. Primarily, the unique characteristics of AI technologies, being fundamentally different from that of ordinary scientific and technological innovations, enable end-users’ opportunities of active participation, rather than passive consumption. Further, in the participation process, AI transcends being merely a technological tool but rather assumes the role of a partner to human end-users [38], not to mention that these unprecedented characteristics of AI are taking place against the backdrop of rapid advancements in the field. These considerations indicate that understanding how AI operates may act as one of the key determinants in shaping how people engage with and evaluate AI systems. In a study similar to ours, Ho and colleagues examined the predictive values of domain-specific, objective knowledge and heuristic cues in understanding public acceptance of AI-powered autonomous vehicles and found non-significant associations between knowledge and willingness to use when various heuristic cues were considered [24]. One of our objectives in this study is thus an attempt to replicate and validate this finding in an emerging AI context as we gauge public opinion toward the more novel application of autonomous delivery drones.

#### The role of knowledge.

Empirically, some studies seem to suggest that knowledge could play a significant role in predicting AI acceptance by extending existing theoretical models with actual or perceived knowledge as predictors [39]. For example, Gado and colleagues reported that perceived AI knowledge was predictive of use intention [15]. Similarly, Chiu and colleagues observed that perceived AI knowledge moderated the relationship between adverse AI outcome anticipation and attitude, that is, people with greater subjective knowledge were less likely to be negatively impacted by perceived threats [13]. Al Omari and colleagues observed in their large-scale public survey across multiple Arab countries that greater objective knowledge of AI was indicative of the intention to use medical AI technologies [40]. Tan and colleagues reported that objective knowledge of autonomous vehicles was indicative of support [16]. Si found similar findings in South Korea in that a broader knowledge construct, AI literacy, was associated with attitudes toward AI and use intention [41]. Schiavo and colleagues revealed AI literacy was associated with AI acceptance and negatively associated with anxiety [14]. There has been increasing scholarly attention on the effects of AI literacy, with growing calls for more comprehensive education [42], as well as more effective operational definitions encompassing nuanced and localized measurements [43]. Following Aydin, we set out to assess objective knowledge through pre-defined dimensions including, application, history, electronic and mechanical structure, software programming, as well as laws and regulations [44]. We set out to measure objective knowledge toward autonomous delivery drones with factors that capture domain-specific knowledge. In doing so, our knowledge factor captures objective understanding of the current status of drone operation and development, potential risk events, and protection frameworks. Based on emerging reports, we propose the following hypothesis for examination:

**H1**: Knowledge positively predicts support for AI-powered autonomous delivery drones.

#### The role of heuristics.

In the trust in AI scholarship, the trust in automation framework [45] has received extensive recognition. The framework delineates three primary trust factors as attitudinal evaluations toward automation, namely dispositional, situational, and learned trust. Hoff and Bashir defined *dispositional trust* as “an individual’s overall tendency to trust automation, independent of context or a specific system… [and] refers to long-term tendencies arising from both biological and environmental influences” (p. 413), *situational trust* as a state that “depends on the specific context of an interaction” (p. 413) and “varies greatly depending on the situation” (p. 415), and *learned trust* as “an operator’s evaluations of a system drawn from past experience or the current interaction… [and] is directly influenced by the operator’s preexisting knowledge and the automated system’s performance” (p. 420) [46]. Indeed, a significant body of research has deployed the framework in studying trust in AI and related applications [47,48].

From this perspective, we set out to study the construct of propensity to trust in AI, conceptualized as a dispositional trait that predicts support. Conceptually speaking, trust is “a mental state that the trustor holds toward the trustee with respect to the performance of a behavior in uncertainty and vulnerability” (p. 3) [10]. With trust placed in AI, the mental state involves trust processes similar to those placed in human beings [49]. The predisposition to trust captures the inherent tendency to place trust in AI, and in this study, represents the first and primary construct in the cognitive miser approach. There have been some empirical observations suggesting the predictive value of propensity to trust in determining AI acceptance. For example, Montag and colleagues reported that it reduced fear and increased acceptance of various types of AI applications [50]. Revillod similarly reported that propensity to trust in AI enhanced fairness and usefulness perceptions through higher levels of trust in AI [51]. Yi and colleagues further reported that autonomous vehicle drivers with greater trust propensity tended to be less sensitive to poor system reliability, which buffered trust erosion [52]. We seek to extend this line of research by examining the predictive value of propensity to trust in AI as a cognitive heuristic on support for the specific AI application of autonomous delivery drones, and we propose the following hypothesis for examination:

**H2**: Propensity to trust in AI predicts support for AI-powered autonomous delivery drones.

In the introduction of novel scientific and technological innovations, news media reporting can be of significant influence. The news media may be the primary, or the only, source of information for people to learn about emerging technologies. Indeed, the public’s exposure to drone information predominantly took place through mainstream news media [44,53]. Much research has conceptualized the use of news media as news media *exposure*, referring to contact with news media and is generally operationalized as self-reported behavioral measurements such as time spent on news media consumption; or news media *attention*, referring to the state of increased mental effort spent on news information and is similarly operationalized as self-reported evaluations such as the amount of attention paid [54,55]. A large body of research has shown that news media use was generally indicative of public attitudes toward AI. For example, Brewer and colleagues reported that news media use was associated with attitude of and support for AI [56]. Cui and Wu similarly reported that following AI-related information and discussions on news media was associated with benefit perception in and policy support for AI [57]. Ho and Cheung reported the news media attention toward autonomous passenger drones was positively related to use intention [10]. Cheung and Ho further reported that news media attention to AI in general was associated with public support through explainability perception and trust in AI [8]. Importantly, Ho and Cheung emphasized the importance of framing and reporting tone in the interpretation of findings [10], that is, favorable attitudinal outcomes and higher levels of public acceptance were only associated with positive, or at the least neutral, news media coverage. Indeed, different reporting frames on AI were prevalent and could influence public perceptions in different directions [58]. Thus, we performed a content analysis on news articles published in the Singapore print media. We extracted all news articles on autonomous delivery drones published by The Straits Times, Business Times Singapore, and Singaporean Government News from Factiva. The search syntax included delivery drone OR drone delivery AND Singapore. The search covered news articles published in the past 5 years prior to 17th May 2023. This resulted in 39 unique news articles. Two independent coders analyzed the tone of the articles (inter-coder reliability: r = 1.00). The news articles content analysis revealed that the reporting tone concerning delivery drones was predominantly characterized by a neutral or positive tone. As such, we propose the following hypothesis for examination:

**H3**: News media attention predicts support for AI-powered autonomous delivery drones.

A heuristic cue closely linked to news media attention is the credibility perception of the information source. Westerman and colleagues recognized that source credibility perception has in recent years received more scholarly attention as the amount of information available in new forms of news media substantially increased [59]. Perceived source credibility (i.e., sometimes referred to as perceived credibility), conceptually speaking, “consists of the judgements made by a perceiver (e.g., a message recipient) concerning the believability of a communicator” (p. 181) [60]. Operating as a peripheral cue, people opt to rely on the expertise of the source, instead of the content of the information, to make judgements on the issue of interest [61]. While Aydin offered an exploration of how the public could acquire knowledge about drones [44], which focused particularly on sources of information with an emphasis on news media outlets, our objective is to venture beyond and investigate the impact of credibility perception of various information sources on support. Here, we focus on three sources of information that represent, as we view them, the most relevant stakeholders of autonomous delivery drones, namely scientists, authorities, and business leaders in the autonomous delivery drone development field. Taken together, we raise the following research question:

**RQ1**: How do perceived source credibility of (a) scientists, (b) authorities, and (c) business leaders predict support for AI-powered autonomous delivery drones?

Another widely recognized heuristic cue in the field of science and technology pertains to the perceptions of risk and benefit inherent in technological innovations. Risk perception refers to the subjective internal evaluation of hazardous events and subject matters [62], whereas benefit perception is viewed as the subjective internal assessment of positive value associated with the object of interest [63,64]. From this perspective, a large body of research has indicated that the public developed acceptance toward novel technological innovations based on perceived risks and benefits, including, but are not limited to, those associated with gene technology [65], nanotechnology [22], and autonomous vehicles [24,66]. Based on the substantial evidence as presented in the literature, we propose the following hypotheses for examination:

**H4**: Risk perception negatively predicts support for AI-powered autonomous delivery drones.
**H5**: Benefit perception positively predicts support for AI-powered autonomous delivery drones.

#### A comparison of relative weights.

While it has been rather firmly established that cognitive heuristics could significantly impact technological acceptance, regardless of the influence of scientific knowledge, how each heuristic cue performs in relation to each other has received much less attention. One example is when Ho and colleagues reported meta-analytic findings with effect sizes on various explanatory variables such as risk/benefit perceptions, cost perception, and trust [23]. We note that findings such as these can provide global evidence that explain the effectiveness of each explanatory variable across studies, but they generally do not illuminate each variable’s relative importance (i.e., also referred to as relative weight), that is, the proportion of variance in the dependent variable explained by each predictor taking into account both their distinct as well as shared variance. Examining relative importance would enable us to make precise observations on how well each explanatory variable in the model contributes to the dependent variable, thereby allowing us to reveal more in-depth practical insights. Indeed, some studies have examined relative importance of antecedents determining technology acceptance to shed light on theory [67]. We thus set out to examine the relative weights of each cognitive heuristic and raise the following research question:

**RQ2**: What is the relative importance of various cognitive heuristics in explaining support for AI-powered autonomous delivery drones?

## Method

### Procedure

We commissioned a research company to collect online survey data between 28^th^ April and 17^th^ May, 2023. Inclusion criteria of the samples were: 1. Singaporean or permanent resident, and 2. aged 21 or above. To ensure representativeness, sampling was stratified to match the Singaporean census quotas by age, gender, and ethnicity. The data analyzed in the present study is a sub-set of a large-scale public opinion survey on AI and autonomous drones in Singapore. All respondents provided written informed consent prior to responding to the survey. The public opinion survey underwent a cognitive testing interview procedure [68], in which several individuals with profiles matching the target sampling profiles were asked to evaluate the questions, instruction, conceptual definitions, items, and survey design choices. This procedure enabled us to preemptively address erroneous perceptions which may be held by the respondents, thereby reducing response error and enhancing reliability. The public survey was administered in English. A separate portion of the public survey examined a similar AI application which findings have been published elsewhere [[8,10] [69]].

Before respondents evaluated items related to autonomous delivery drones, they were required to stay on a concept page for at least 30 seconds. The page showed the definition of AI-powered autonomous delivery drones (i.e., “Autonomous delivery drones” are vehicles in the air which can operate fully autonomously with artificial intelligence (AI) technology and without human intervention in executing the flight mission for the purpose of goods delivery from location to location. In other words, it is a “self-flying drone” for goods delivery based entirely on algorithms from its associated artificial intelligence (AI) system), as well as a brief introduction of AI. An image was also shown at the bottom of the page to ensure consistent comprehension. Subsequently, all items were measured on a 5-point Likert/Likert-type scale, except for the knowledge scale of which the options “true,” “false,” and “do not know” were provided.

### Respondents

We removed respondents who were unable to 1. pass at least two out of three attention checks, and 2. complete the survey within a reasonable time. Consequently, a total of 1,002 respondents were retained, of which 910 were Singaporeans (90.8%) and 92 were permanent residents (9.2%). On gender, 493 were men (49.2%) and 509 were women (50.8%). On ethnicity, the respondents were predominantly Chinese (*n* = 746; 74.4%), followed by Malay (*n* = 140; 14.0%), Indian (*n* = 85; 8.5%), and others (*n* = 31; 3.1%). There were 159 respondents aged between 21 and 29 (15.9%), 176 aged between 30 and 39 (17.6%), 184 aged between 40 and 49 (18.4%), 195 aged between 50 and 59 (19.5%), 89 aged between 60 and 64 (8.9%) and 199 aged 65 or above (19.9%). On educational level, 7 did not receive formal education (.7%), 7 completed primary education (.7%), 44 completed secondary education (4.4%), 47 completed N-level (4.7%), 88 completed O-level (8.8%), 41 completed A-level (4.1%), 228 obtained diploma and professional qualifications (22.8%), 423 obtained bachelor’s degree (42.3%), 102 obtained master’s degree (10.2%), and 15 obtained doctoral degree (1.5%).

### Instruments

*Propensity to trust in AI*. Propensity to trust in AI was measured by an adapted five-item 5-point Likert scale asking respondents to rate their dispositional tendency to trust AI systems [70,71]. Higher scores reflected higher levels of trust (*M* = 3.14; *SD* = .76). The scale presented satisfactory internal reliability (Cronbach’s α = .90).

*News media attention in AI-powered autonomous delivery drones*. News media attention was measured by a self-developed four-item 5-point Likert-type scale asking respondents to rate their levels of news media attention in autonomous delivery drones across four platforms (i.e., TV news, print news, online news, and social media). Higher scores reflected higher attention (*M* = 2.92; *SD* = .98). The scale presented satisfactory internal reliability (Cronbach’s α = .88).

*Knowledge of AI-powered autonomous delivery drones*. Knowledge was measured by a self-developed ten-item 3-point scale asking respondents to evaluate statements related to autonomous delivery drones by stating true, false, or I do not know. The ten items were developed based on established dimensions [44], namely application, history, electronic and mechanical structure, software programming or coding, as well as laws and regulations of autonomous delivery drones. The items were co-developed with engineering scientists working on autonomous drones in Singapore, with the intention to gauge objective understanding of autonomous delivery drones. Subsequently, correct responses were coded as 1 whereas incorrect and “I do not know” responses were coded as 0. Sum scores were computed in which higher scores reflected better knowledge (*M* = 3.66; *SD* = 2.01).

*Perceived source credibility*. Perceived source credibility was measured by an adapted five-item 5-point Likert-type scale asking respondents to rate their trust levels in the information given by the sources of scientists (i.e., university and industry scientists), authorities (i.e., the Singaporean government and international institutions), and business leaders (i.e., business leaders in autonomous delivery drone industry) regarding the risks and benefits of autonomous delivery drones [72]. Higher scores reflected higher levels of trust in the source (scientists: *M* = 3.56; *SD* = .71; authorities: *M* = 3.69; *SD* = .76; business leaders: *M* = 3.24; *SD* = .87). The scales presented satisfactory internal reliability (Scientists: Cronbach’s α = .77; Authorities: Cronbach’s α = .74).

*Risk perception of AI-powered autonomous delivery drones*. Risk perception was measured by an adapted seven-item 5-point Likert scale asking respondents to rate their perception of risks of autonomous delivery drones [73]. Higher scores reflected higher levels of risk perception (*M* = 3.63; *SD* = .58). The scale presented satisfactory internal reliability (Cronbach’s α = .85).

*Benefit perception of AI-powered autonomous delivery drones*. Benefit perception was measured by an adapted five-item 5-point Likert scale asking respondents to rate their perception of benefits of autonomous delivery drones [73]. Higher scores reflected higher levels of benefit perception (*M* = 3.52; *SD* = .57). The scale presented satisfactory internal reliability (Cronbach’s α = .77).

*Support for AI-powered autonomous delivery drones*. Support was measured by a self-developed three-item 5-point Likert scale asking respondents to rate their support for the development, deployment, and government funding of research of autonomous delivery drones in Singapore. Higher scores reflected higher levels of support (*M* = 3.65; *SD* = .79). The scale presented satisfactory internal reliability (Cronbach’s α = .91).

Exact item wordings and descriptive statistics are presented in Table 1.

**Table 1:** Exact Item Wording and Descriptive Statistics.

Item	*M*	*SD*	Cronbach’s α
**Propensity to trust in AI**	3.14	.76	.90
*On a scale of 1–5 (1 = Strongly disagree, 5 = Strongly agree), to what extent do you agree or disagree with the following statements?*			
Unless there is a reason not to, I usually trust artificial intelligence systems.			
In general, I would take the help of artificial intelligence systems.			
I have a strong tendency to trust artificial intelligence systems.			
It is easy for me to put my faith in artificial intelligence systems.			
Even if I have limited knowledge about an artificial intelligence system, I am likely to trust it.			
**News media attention in AI-powered autonomous delivery drones**	2.92	.98	.88
*On a scale of 1–5 (1 = No attention at all, 5 = A lot of attention), how much attention do you generally pay to … about autonomous delivery drones?*			
TV news			
Print news			
Online news			
Social media			
**Knowledge of AI-powered autonomous delivery drones**	3.66	2.01	–
*Please indicate whether you think each statement listed below is true or false. If you are not sure, please mark ‘not sure’ (do not guess!).*			
Autonomous delivery drones are used to deliver pizzas. (T)			
Autonomous delivery drones cannot be used to deliver vaccines. (F)			
Autonomous delivery drones came about in the early 2000s. (F)			
Autonomous delivery drones have been tested in the United States. (T)			
Autonomous delivery drones are often equipped with a camera. (T)			
It is necessary to minimize power consumption in information processing on autonomous delivery drones. (T)			
Most of the information processing is performed on board of the autonomous delivery drone. (T)			
The programming of autonomous delivery drones allows them to change a flight destination if necessary. (F)			
It is legal to use autonomous delivery drones as a mode of goods delivery in Singapore. (F)			
When an accident occurs with autonomous delivery drones, it is clear which party will bear the responsibility under Singaporean law. (F)			
**Perceived source credibility (Scientists)**	3.56	.71	.77
*On a scale of 1–5 (1 = Do not trust at all, 5 = Trust very much), how much do you trust the information given by the following sources regarding the risks and benefits of autonomous delivery drones?*			
University scientists (i.e., academics/ researchers) doing research in autonomous delivery drones			
Industry scientists (i.e., engineers/ programmers) working for autonomous delivery drone companies			
**Perceived source credibility (Authorities)**	3.69	.76	.74
*On a scale of 1–5 (1 = Do not trust at all, 5 = Trust very much), how much do you trust the information given by the following sources regarding the risks and benefits of autonomous delivery drones?*			
The Singaporean government			
International institutions (e.g., International Civil Aviation Organization, International Air Transport Association)			
**Perceived source credibility (Business leaders)**	3.24	.87	–
*On a scale of 1–5 (1 = Do not trust at all, 5 = Trust very much), how much do you trust the information given by the following source regarding the risks and benefits of autonomous delivery drones?*			
Business leaders in autonomous delivery drone industry			
**Risk perception of AI-powered autonomous delivery drones**	3.63	.58	.85
*On a scale of 1–5 (1 = Strongly disagree, 5 = Strongly agree), to what extent do you agree or disagree with the following statements?*			
Autonomous delivery drones may cause safety consequences triggered by technical error.			
Autonomous delivery drones may be confused in unexpected/ unprecedented situations.			
Autonomous delivery drones may not be as safe as human drivers on delivery trucks.			
Autonomous delivery drones may not be secure from hackers.			
Autonomous delivery drones may lead to privacy issues caused by steady tracking of exact location.			
Autonomous delivery drones may lead to job losses.			
Autonomous delivery drones may be dangerous while there are also human-operated drones (e.g., civilian photography drones in the sky).			
**Benefit perception of AI-powered autonomous delivery drones**	3.52	.57	.77
*On a scale of 1–5 (1 = Strongly disagree, 5 = Strongly agree), to what extent do you agree or disagree with the following statements?*			
Autonomous delivery drones may be safer than manual flying (fewer fatal crashes, better response in emergency situations).			
Autonomous delivery drones may have less energy consumption than conventional delivery methods.			
Autonomous delivery drones may lead to less traffic jams.			
Autonomous delivery drones may yield shorter delivery times.			
Using autonomous delivery drones may give me social recognition (image/ reputation)			
**Support for AI-powered autonomous delivery drones**	3.65	.79	.91
*On a scale of 1–5 (1 = Strongly disagree, 5 = Strongly agree), to what extent do you agree or disagree with the following statements?*			
I support the development of autonomous delivery drones in Singapore.			
I support the deployment of autonomous delivery drones in Singapore.			
I support government funding of research in autonomous delivery drones in Singapore.			

### Data analyses

All data analyses were performed on SPSS 26.0 and R-4.3.1. Study variables had been mean centered for the regression analyses to simplify the interpretation of coefficient values. The mean-centered variables were entered into the regression equation based on an assumed causal order, a practice consistently carried out in previous empirical studies [24,36]. More concretely, control variables were entered in the first block, followed by the dispositional variable of propensity to trust in AI, the informational variable of news media attention, and knowledge (being a likely outcome of news media attention), as well as internal perceptions of source credibility perceptions, and finally risk and benefit perceptions. Subsequently, we estimated relative importance indices of the predictor variables [74] with 1,000 bootstrap resamples using the R package relaimpo [75]. Relative importance indices have been advocated in social sciences research as an appropriate supplement to regression analyses, as it offers to separate explained variance among predicting variables to offer precise observations on relative importance [74].

## Results

### Correlations

Correlations of study variables presented relationships consistent with the hypotheses. More concretely, support was positively correlated with all variables, except for risk perception (*p* = .081). The zero-order correlation matrix is presented in Table 2.

**Table 2:** Zero-order Correlation Matrix of Study Variables.

Variable	1	2	3	4	5	6	7	8	9
1. Trust propensity	–								
2. News media attention	.39***	–							
3. Knowledge	.19***	.33***	–						
4. Source credibility (Scientists)	.46***	.40***	.22***	–					
5. Source credibility (Authorities)	.45***	.38***	.24***	.70***	–				
6. Source credibility (Business leaders)	.45***	.36***	.22***	.62***	.53***	–			
7. Risk perception	−.003	.07*	.08*	.15***	.16***	.09**	–		
8. Benefit perception	.44***	.44***	.28***	.58***	.57***	.45***	.27***	–	
9. Support	.46***	.43***	.25***	.59***	.60***	.42***	.06	.59***	–

*Note*. *N* = 1,002.

*** *p* < .001, ** *p* < .01, * *p* < .05.

### Regression model and relative weight analyses

The regression model accounted for 52.20% of the variance in support for AI-powered autonomous delivery drones. Hypothesis 1 suggests knowledge predicts support for AI-powered autonomous delivery drones. Although block 4 containing knowledge contributed to an incremental *R²* of.008 (*p* < .01), knowledge did not consequently predict support (β = .02, *p* = .349; Model 6). Thus, H1 was not supported. Hypothesis 2 suggests that propensity to trust in AI predicts support for AI-powered autonomous delivery drones. Our results revealed that propensity to trust in AI positively predicted support (β = .11, *p* < .001; Model 6). Relative weight analysis showed that propensity to trust in AI predicted 5.45% in support (95% CI = .07%, 1.20%, *p* < .05). Thus, H2 was supported. Hypothesis 3 suggests that news media attention predicts support for AI-powered autonomous delivery drones. Our analyses revealed that news media attention positively predicted support (β = .11, *p* < .001; Model 6). Relative weight analysis showed that news media attention predicted 4.88% in support (95% CI = 3.44%, 6.78%; *p* < .05). Thus, H3 was supported. Research Question 1 asks how perceived source credibility of (a) scientists, (b) authorities, and (c) business leaders predict support for AI-powered autonomous delivery drones. Answering RQ1, perceived source credibility of scientists positively predicted support (β = .21, *p* < .001; Model 6). Relative weight analysis showed that perceived source credibility of scientists predicted 10.75% in support (95% CI = 8.40%, 13.19%; *p* < .05). Similarly, perceived source credibility of authorities positively predicted support (β = .24, *p* < .001; Model 6). Relative weight analysis showed that perceived source credibility of authorities predicted 11.79% in support (95% CI = 9.49%, 14.35%; *p* < .05). Perceived source credibility of business leaders did not predict support (β = −.05, *p* = .096; Model 6). Subsequently, Hypotheses 4 and 5 suggest that risk and benefit perceptions negatively and positively predict support for AI-powered autonomous delivery drones. Our analyses revealed that risk perception negatively predicted support (β = −.09, *p* < .001; Model 6). Relative weight analysis showed risk perception predicted.51% in support (95% CI = .25%, 1.22%; *p* < .05). Thus, H4 was supported. Similarly, benefit perception positively predicted support (β = .29, *p* < .001; Model 6). Relative weight analysis showed benefit perception predicted 12.42% in support (95% CI = 9.35%, 15.71%; *p* < .05). Thus, H5 was supported. Research Question 2 asks what the relative importance of various cognitive heuristics in explaining support for AI-powered autonomous delivery drones would be. Answering RQ2, benefit perception carried the most weight in explaining support (12.42% [95% CI = 9.35%, 15.71%]), followed by perceived source credibility of authorities (11.79% [95% CI = 9.49%, 14.35%]), perceived source credibility of scientists (10.75% [95% CI = 8.40%, 13.19%]), propensity to trust in AI (5.45% [95% CI = 3.74%, 7.68%]), news media attention (4.88% [95% CI = 3.44%, 6.78%]), perceived source credibility of business leaders (3.51% [95% CI = 2.55%, 4.75%]), and finally risk perception (.51% [95% CI = .25%, 1.22%], in this order. Results with standardized coefficient estimates are presented in Table 3.

**Table 3:** Ordinary Least Squares Hierarchical Regression Analyses on Factors Predicting Support for AI-powered Autonomous Delivery Drones.

Variables	Model 1 β	Model 2 β	Model 3 β	Model 4 β	Model 5 β	Model 6 β	RW [95% CI]
**Block 1: Control variables**							
Gender	−.10**	−.10***	−.07*	−.06*	−.04	−.05	.44% [.07%, 1.20%]
Age	−.01	.04	.04*	.03	.01	.004	.06% [.03%,.34%]
Ethnicity	−.04	−.02	−.03	−.03	−.03	−.04	.14% [.02%,.88%]
Prior use of drones	−.07*	.01	.09**	.09***	.04	.04	.22% [.14%,.55%]
Educational level	.07*	.04	.02	.01	.01	.01	.23% [.07%,.90%]
Marital status	.03	−.01	−.02	−.02	.01	.01	.04% [.02%,.42%]
Type of residence	.09**	.07*	.05	.05	.04	.05	.50% [.12%, 1.31%]
**Block 2: Dispositional trait**							
Propensity to trust in AI		.45***	.35***	.34***	.15***	.11***	5.45% [3.74%, 7.68%]
**Block 3: News media attention**							
News media attention in AI-powered autonomous delivery drones			.31***	.29***	.16***	.11***	4.88% [3.44%, 6.78%]
**Block 4: Knowledge**							
Knowledge of AI-powered autonomous delivery drones				.10**	.04	.02	1.25% [.63%, 2.30%]
**Block 5: Source credibility perception**							
Perceived source credibility (Scientists)					.27***	.21***	10.75% [8.40%, 13.19%]
Perceived source credibility (Authorities)					.29***	.24***	11.79% [9.49%, 14.35%]
Perceived source credibility (Business leaders)					−.04	−.05	3.51% [2.55%, 4.75%]
**Block 6: Internal perceptions**							
Risk perception						−.09***	.51% [.25%, 1.22%]
Benefit perception						.29***	12.42% [9.35%, 15.71%]
***F***	5.66*** (7, 994)	37.29*** (8, 993)	47.78*** (9, 992)	44.50*** (10, 991)	69.44*** (13, 988)	71.77*** (15, 986)	
***∆R²***	.038***	.193***	.071***	.008**	.168***	.045***	
***Total R²***						.522	
***Total adjusted R²***						.515	

*Note*. RW = Relative weights in percentage of *R²* explained.

*** *p* < .001, ** *p* < .01, * *p* < .05.

### Mediation model

**Fig 1:**
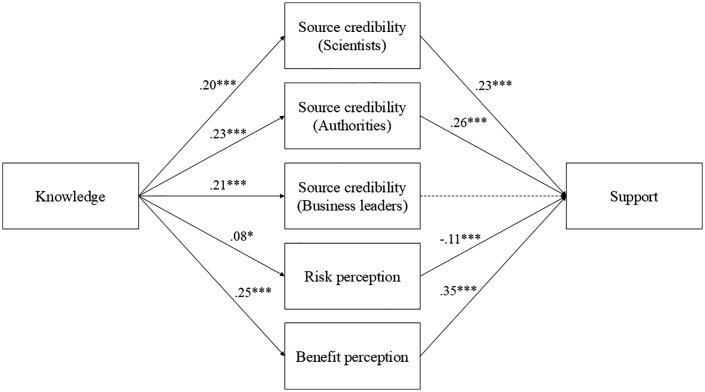
Mediation Model Illustrating Indirect Effects of Knowledge of AI-powered Autonomous Delivery Drones on Support through Source Credibility Perceptions (i.e., Scientists, Authorities, and Business Leaders), Risk Perception, and Benefit Perception, Accounting for Gender, Age, Ethnicity, Education, Marital Status, Type of Residence, and Prior Use of Drone. Coefficient estimates are standardized. *** *p* < .001, ** *p* < .01, * *p* < .05.

As observed in Table 3, the association between knowledge and support was undermined by the presence of additional heuristic cues (i.e., source credibility perceptions and risk/benefit perceptions). As the predictive power of knowledge was accounted for by the inclusion of the entered variables in Models 5 and 6, we then speculated that knowledge operated indirectly through heuristic cues to determine support. Thus, a mediation model was further estimated using Model 4 of the PROCESS macro, version 4.2 [76] on SPSS with knowledge of AI-powered autonomous delivery drones as independent variable, support as dependent variable, and source credibility perceptions of scientists, authorities, and business leaders, as well as risk and benefit perceptions as mediating variables, using 5,000 bootstrap resamples. Results revealed significant indirect effects through all mediators except source credibility perception of business leaders, 95% CI [−.007,.003]. The indirect effect through source credibility perception of scientists, *B* = .02, 95% CI [.011,.027], accounted for 26.45% of the total effect. The indirect effect through source credibility perception of authorities, *B* = .02, 95% CI [.015,.033], accounted for 33.24% of the total effect. The indirect effect through risk perception, *B* = −.004, 95% [−.007, −.001], accounted for 5.20% of the total effect. The indirect effect through benefit perception, *B* = .033, 95% CI [.024,.044], accounted for 47.54% of the total effect. The direct effect of knowledge on support was non-significant, 95% CI [−.004,.034], suggesting a pattern consistent with full mediation effects. In this mediation model, the sum of the proportion mediated exceeded the total effect, which can be a result of mediators exerting influence on each other, thereby creating overlapping mediation paths, or because the model included negative proportion mediated (i.e., risk perception). This phenomenon is not uncommon in models with multiple mediators [77]. The mediation model with standardized coefficient paths is illustrated in Fig 1.

## Discussion

Of principal concern in the present study was the investigation into whether knowledge and heuristic cues were indicative of AI support. In resolving theoretical conflicts between models of knowledge deficit and cognitive miser, our data suggests that knowledge in the application of autonomous delivery drones, while initially relevant, was not predictive of support when heuristic variables such as perceived source credibility and risk/benefit perceptions were subsequently entered into the model. Thus, findings in the present study were consistent with prior research in which the cognitive miser model generally prevailed in various technoscientific areas [23,25]. Overall, these observations suggest that people generally relied on mental shortcuts when pondering upon AI acceptance, and contradicted emerging evidence suggesting that AI knowledge could motivate people to accept AI technologies.

Does this mean that knowledge has no value, whatsoever? Not so. From our data, it seems clear that knowledge was indicative of support, at least to a certain extent (i.e., Model 4). Its role diminished only when heuristics with higher levels of relative weights were explicitly taken into account. The subsequent mediation model likewise showed that knowledge exerted an indirect effect on support through various heuristic cues. Indeed, even though overwhelming reports present the strength of the cognitive miser approach in determining technological support, our findings could be viewed as an indication that, on a theoretical level, knowledge deficit and cognitive miser models may co-exist rather than being mutually exclusive, at least to a certain extent. Viewed in this lens, knowledge (i.e., in the objective dimension) remains theoretically and empirically relevant as AI continues to develop and further integrate into our society. Indeed, in the fast-emerging AI research field, there has been significant traction in studying AI literacy [13,42,43,78,79]. We note that the conceptual and operational definitions of knowledge in the present study reflected a specific type of knowledge, that is, application-level understanding. This view may be rather limited in scope as compared to literacy, which can encapsulate broader factors such as *awareness*, the capacity to recognize and understand applications; *usage*, the practical ability to operate applications; *evaluation*, the competence to critically examine functionalities; and *ethics*, the ability to judge ethical risks and responsibilities [80]. We encourage future studies to investigate how AI literacy and its underlying dimensions are associated with internal and behavioral outcomes, and more importantly, across applications, to offer nuanced theoretical and practical insights. We note, however, that in the present study, objective knowledge about autonomous delivery drones remained on the lower end. Mean and standard deviation suggest that knowledge levels remained at a restricted range with a majority of the respondents answering correctly in one through six items. While this may be a reflection that the public has not fully registered what the autonomous delivery drone technology entails, the positively skewed measurement scale suggests a floor effect which could potentially constrain the detection of a significant linear relationship. In hindsight, we recognize that some technical items may have presented challenges to laypeople. We encourage future studies to continue monitoring objective knowledge levels and make necessary refinements to our measurement.

Our observations painted a rather clear picture on the robustness of heuristic cues in determining AI support. Foremost, our focus on informational heuristics offered the opportunity to investigate whether news media could shape public opinion on AI applications. Consistent with prior research, news media attention in AI presented itself as a robust predictor of AI acceptance [8]. While not being as important as other variables in terms of relative weights, it remained stable across all regression models, providing strong support that news media plays an essential role in introducing emerging technologies [81]. It becomes rather intriguing when this effect is contrasted with the non-significant relationship between knowledge and support because, in theory, news media attention and knowledge should strongly correlate and therefore present predictive power similar in nature [55]. Examining our finding in this lens adds to the discussion that contrasts attention and knowledge. For example, Ho and colleagues found that news media attention to nuclear energy did not predict support while domain-specific, objective knowledge did [37]. Later, in understanding public opinion of alternative aquafeed, Ho and colleagues reported that neither news media attention nor objective knowledge predicted support [25]. In the context of autonomous vehicles, however, a pattern similar to the present study’s emerged [24]. Even though measurement variations exist, these prior public opinion reports appear to suggest that the predictive values of these variables may be dependent on the technoscientific context in question. If so, one speculation is that technologies or scientific issues that are novel and opaque in nature may benefit more through news media attention than objective knowledge levels. When they present threats that are psychologically proximate, particularly, people may opt to rely on heuristics such as news media narratives to make sense of the threat, whereas when the threat is perceived as more distant, they may be motivated to rely on rational decision-making. In the case of aquafeed, however, neither process is engaged presumably because the issue lacks personal relevance in comparison. We encourage future studies to investigate the nuances between attention and knowledge in various technoscientific contexts, preferably through the perspective of construal level theory, which suggests that mental construal of objects depend on how psychologically distant that object is perceived to be, for further theorization [82].

Our results suggest, subsequently, that not all information sources in the news media were equally effective, that is, the public appeared to find information provided by scientists and authorities credible but discounted that by business leaders. While perceived source credibility of scientists was not entirely unexpected, an interesting observation is the substantial value placed in the credibility of authorities. Relative weight analyses revealed that perceived source credibility of authorities not only carried greater weight than that of scientists, but that it was the second most important contributor in the model. This is likely an indication of public trust toward the government in science communication. Indeed, the Singaporean government has consistently been rated a highly trusted institution in the city-state [83]. A plausible explanation on observations regarding business leaders may be that the public perceives them to act primarily on commercial interests, and for that reason the information they convey may not carry as much weight. Indeed, Goh and Ho identified a theoretical framework delineating the dimensions of trustworthiness on AI technology developers [84], in which the public reported expected trustworthiness factors such as accountability, objectiveness, and transparency, all of which tend to contradict self-serving motivations. If information must come from the commercial sector, meta-analytic evidence suggests that temporally delaying source identification may counteract some negative effects of poor source credibility [61], thereby facilitating a more objective reception of the information. Science communicators may tailor communication strategies accordingly. Nevertheless, we note that our findings in Singapore may not necessarily generalize in geographical contexts that exhibit neither high levels of institutional trust nor regulated news media narratives. Indeed, Chen and Wen’s study reveals that trust in government was positively associated with both trust in AI and trust in AI science community [85], suggesting that trust in authorities could play a significant role in determining support. Thus we contend that in locations where governmental or scientific authorities are not perceived as trustworthy stewards of AI technologies, we should not expect the same predictive value of trust in institutional social actors in determining AI support as observed here. Replication studies in other parts of the world will offer nuanced comparisons.

We observed some unexpected findings in relative weights of risk and benefit perceptions. Despite that the block containing these perceptions contributed a noteworthy incremental *R²* of.168, relative weight analyses revealed that benefit perception played a considerably greater role than risk perception. We suspect this may reflect the fact that autonomous delivery drones are not yet widely introduced in the Singaporean society, and therefore potential risks may not be a salient factor. If this was not the case, it may reflect a concerning reality in which people appeared to dismiss the risks inherent in AI, and would stand in sharp contrast with observations in other geographical locations such as the U.S., in which the public perceived delivery drones to introduce risks such as system malfunction, privacy intrusion, personal injury, property destruction, and more [86]. We also note that risk and benefit perceptions were not inversely correlated as literature suggests [87,88]. The Singaporean public may simultaneously evaluate AI-powered autonomous delivery drones to be beneficial and risky, and how this ambivalent opinion should evolve as these drones continue to integrate into society deserves scholarly attention. We encourage future studies to continue examining public opinion and make comparisons accordingly. Practically speaking, because source credibility perceptions carried substantial weight, it is essential to ensure that the public places trust in key stakeholders that supply information about AI. We note that even though news media framing can be influential to AI acceptance, if such narratives entail persuasion processes that are perceived as manipulative, it can be counterproductive in the long term as such rhetoric will likely undermine public confidence ininformation source.

In analyzing our data, we further examined potential mediation effects between knowledge and support through heuristic cues. Indirect effect analyses suggest that source credibility perceptions of scientists and authorities, as well as risk and benefit perceptions served as mediating variables. Thus, even though knowledge did not directly predict AI support, its value should not be overlooked. That knowledge exerts an indirect effect on outcome measures and internal variables is empirically aligned with some existing reports. Robinson-Tay and Peng, for example, revealed that objective knowledge of autonomous vehicles was associated with risk perception through trust [89]. Notably, Zhang and Liu directly compared the predictive values of subjective and objective knowledge of genetically modified foods and found that only objective knowledge exerted direct influence on risk and benefit perceptions, which then led to acceptance outcomes [90]. While objective knowledge may present some operationalization challenges (i.e., compared to subjective knowledge), especially when the technoscientific subject matter is of novel nature, it may be worthwhile for future studies to develop and adapt measures to capture this variable to ensure a more comprehensive view of the public opinion landscape. Ideally, people are motivated to support advanced technologies such as AI because they understand the technology, demonstrating informed decision-making. We thus encourage authorities to dedicate effort to ensure, through different channels, that members of the public gain sufficient objective technological knowledge, or more abstractly, scientific knowledge, in securing support. Even though our results suggest that the effectiveness of cognitive shortcuts may be more robust and direct, we nevertheless contend that technological acceptance bypassing cognitive understanding and rational decision-making may pose ethical challenges. We urge policymakers and science communicators to heed these results and develop appropriate measures accordingly.

Despite our best intentions, our study is not without limitations. First, we note that our data was cross-sectional and so we refrain from causality claims. Second, we note that the self-report nature of our survey variables may result in recall biases and thereby present inaccurate assessments. Third, in the present study news coverage on AI in our context was predominantly neutral and positive, a phenomenon consistent with the Singaporean media landscape [91]. In other parts of the world, AI technologies may be framed divergently in terms of outlook [92]. Future studies are encouraged to study the effectiveness of different types of information frames in determining internal evaluations and behavioral acceptance. Finally, despite our efforts to stratify samples to effectively represent all demographic groups, the nature of online survey inherently prevented certain people from participating, such as older adults who were not technology-savvy.

## Conclusion

As autonomous delivery drones are actively being researched, developed, and introduced in many parts of the world, we set out to gauge public opinion toward the emerging AI technology in the city-state of Singapore. From a theoretical perspective, we contrasted the knowledge deficit model against the cognitive miser model, with results strongly suggesting that the cognitive miser perspective prevailed as AI knowledge was consequently not indicative of public AI support. We also revealed that trust propensity, news media attention in AI, perceived source credibility of scientists and authorities, as well as risk and benefit perceptions predicted public support. Relative weight analyses further illuminated the relative importance of various heuristics in the model. Even though objective knowledge was not directly associated with support, mediation analyses subsequently showed that it exerted indirect influence through various heuristic cues. We thus urge science communicators and policymakers to heed our findings and design appropriate measures to, on one hand, secure informed buy-in of AI technologies through information channels while enhancing credibility perceptions of the information sources and, on the other hand, employ strategic pedagogical means to ensure a reasonable level of AI knowledge in the public. As the technology continues to develop, we encourage future studies to continue monitoring public opinion of AI in other geographical contexts.
